# Supplementary material for: Probiotics for the Prophylaxis of Migraine: A Systematic Review of Randomized Placebo Controlled Trials
Source: J Clin Med. 2019 Sep 11;8(9):1441. doi: 10.3390/jcm8091441 (PMC6780403; doi:10.3390/jcm8091441)
Supplement: Supplementary file 1 [file jcm-08-01441-s001.pdf]

Appendix A

Supplementary material

Table S1. Risk of bias of included studies table [SUPPLEMENTARY DATA].

Bias	De Roos 2017 [1]	Support of Judgment	Martami 2019 [2]	Support of Judgment
Random sequence generation (selection bias)	Low risk	Computer generated, block randomization of 4.	Low risk	Externally performed randomization.
Allocation concealment (selection bias)	Low risk	Allocation was concealed from the researchers and subjects.	Low risk	Allocation was concealed from the researchers and subjects until the final analyses.
Blinding of participants and personnel (performance bias)	Low risk	Externally performed randomization and allocation made it highly unlikely for personnel and participants to discern allocation or adjust randomization. It is unclear when blinding was stopped, whether it was prior or post data analysis.	Low risk	Externally performed randomization and allocation made it highly unlikely for personnel and participants to discern allocation or adjust randomization.
Blinding of outcome assessment (detection bias)	Low risk	Allocation was concealed from the start, however, it is unclear when allocation was uncovered.	Low risk	Allocation was concealed from the start until all subjects had completed the study.
Incomplete outcome data (attrition bias)	Unclear risk	Participants lost to follow-up were listed and their allocation was provided, but no explanation to the withdraw reason was provided.	Low risk	Participants lost to follow-up were listed and their allocation and reason for withdrawal was provided.
Selective reporting (reporting bias)	Low risk	Outcomes in the study protocol were reported in the study.	Low risk	Outcomes in the study protocol were reported in the study.
Other bias	Unclear risk	Modified intention to treat analysis	Low risk	Not specified type of analysis (per protocol or intention to treat).

Table S2. Description of excluded studies (from full text analysis) [SUPPLEMENTARY DATA].

Publication	Reason for Exclusion
de Roos 2015 [3]<br>The effects of the multispecies probiotic mixture Ecologic® Barrier on migraine: Results of an open-label pilot study.	Open label pilot study
Kwong 2016 [4]<br>Oxytocin, a missing link of the gut-brain axis between probiotics and chronic migraine?	Not an original study (comment on another study)
Sensenig 2001 [5]<br>Treatment of migraine with targeted nutrition focused on improved assimilation and elimination.	Different intervention (mixture of a probiotic with peptides and amino acids)
Seyfi-Shahpar, 2017 [6]<br>The effects of probiotic supplementation on chronic migraine (CM) headache: a randomised placebo-controlled double-blind study.	Abstract only
van Hemert 2012 [7]<br>The Effect of Daily Oral Intake of Probiotics on the Frequency and Intensity of Migraine Attacks-A Pilot Study	Abstract only
van Hemert 2014 [8]<br>Migraine Associated with Gastrointestinal Disorders: Review of the Literature and Clinical Implications.	Literature review

References:

1. De Roos, N. M.; Van Hemert, S.; Rovers, J. M. P.; Smits, M. G.; Witteman, B. J. M. The Effects of a Multispecies Probiotic on Migraine and Markers of Intestinal Permeability-Results of a Randomized Placebo-Controlled Study. *Eur. J. Clin. Nutr.* **2017**, *71*, 1455–1462. https://doi.org/10.1038/ejcn.2017.57.
2. Martami, F.; Togha, M.; Seifishahpar, M.; Ghorbani, Z.; Ansari, H.; Karimi, T.; Jahromi, S. R. The Effects of a Multispecies Probiotic Supplement on Inflammatory Markers and Episodic and Chronic Migraine Characteristics: A Randomized Double-Blind Controlled Trial. *Cephalalgia* **2019**, *39*, 841–853. https://doi.org/10.1177/0333102418820102.
3. de Roos, N. M.; Giezenaar, C. G. T.; Rovers, J. M. P.; Witteman, B. J. M.; Smits, M. G.; van Hemert, S. The Effects of the Multispecies Probiotic Mixture Ecologic®Barrier on Migraine: Results of an Open-Label Pilot Study. *Benef. Microbes* **2015**, *6*, 641–646. https://doi.org/10.3920/BM2015.0003.
4. Kwong, K. K.; Chan, S.-T. Oxytocin, a Missing Link of the Gut-Brain Axis between Probiotics and Chronic Migraine? *Beneficial Microbes*. Netherlands November 2016, pp 623–624. https://doi.org/10.3920/BM2016.x003.
5. Sensenig, J.; Marrongelle, J.; Johnson, M.; Staverosky, T. Treatment of Migraine with Targeted Nutrition Focused on Improved Assimilation and Elimination. *Altern. Med. Rev.* **2001**, *6*, 488–494.
6. Seyfi-Shahpar, M.; Martami, F.; Togha, M.; Ghorbani, Z.; Jahromi, S. R.; Ansari, H. 11th European Headache Federation Congress Jointly with 31st Congress of the Italian Society for the Study of Headaches : Rome, Italy. 01-03 December 2017. *J. Headache Pain* **2017**, *18* (1 Supplement 1) (no pagination), 111. https://doi.org/10.1186/s10194-017-0817-z.
7. Van Hemert, S.; Giezenaar, C.; Smits, M. The Effect of Daily Oral Intake of Probiotics on the Frequency and Intensity of Migraine Attacks-A Pilot Study. *Ann. Nutr. Metab.* **2012**, *61*, 334. https://doi.org/http://dx.doi.org/10.1159/000343769.
8. Van Hemert, S.; Breedveld, A.; Rovers, J.; Vermeiden, J.; Witteman, B.; Smits, M. Migraine Associated with Gastrointestinal Disorders: Review of the Literature and Clinical Implications. *Front. Neurol.* **2014**, *5*, 241. https://doi.org/http://dx.doi.org/10.3389/fneur.2014.00241.
